# Resting-state EEG biomarkers of general and domain-specific cognitive impairment and plasma P-tau217 in patients with Alzheimer’s disease and mild cognitive impairment

**DOI:** 10.3389/fneur.2026.1875216

**Published:** 2026-09-07

**Authors:** Liu Yang, Hang Yu, Ze-yu Hong, Zhiyu Liu, Xiao-Jia Li, Fang Ye

**Affiliations:** 1 Department of Neurology, Sichuan Provincial People’s Hospital, University of Electronic Science and Technology of China, Chengdu, China; 2 Department of Neurology, Chengdu Eighth People's Hospital, Chengdu, China; 3 Clinical Research Department, Sichuan Clinical Research Center for Cancer, Sichuan Cancer Hospital and Institute, Sichuan Cancer Center, Affiliated Cancer Hospital of University of Electronic Science and Technology of China, Chengdu, China

**Keywords:** Alzheimer’s disease, cognitive domain, cognitive impairment, plasma biomarker, quantitative EEG

## Abstract

**Background:**

Early detection of Alzheimer’s disease (AD)-related cognitive impairment remains challenging due to the lack of convenient, non-invasive screening tools.

**Objective:**

To explore the multidimensional utility of quantitative electroencephalography (EEG) as a complementary tool for early detection of AD-related cognitive impairment.

**Methods:**

Clinical data, electroencephalogram (EEG) records, cognitive scale scores and blood samples were collected. According to the established clinical diagnostic criteria, the participants were divided into three groups: cognitive normal group, mild cognitive impairment group (MCI) and Alzheimer’s disease (AD) dementia group. Traditional statistical methods and machine learning algorithms were used to analyze the correlation between 200 EEG indexes and the severity of cognitive impairment, domain-specific cognitive impairment and plasma P-tau217 concentration.

**Results:**

A total of 360 participants (154 dementia patients with Alzheimer’s disease, 116 mild cognitive impairment patients and 90 healthy controls) were included. The area under the curve (AUC) of EEG in 360 participants is 0.70–0.87, which can effectively distinguish cognitive dysfunction related to Alzheimer’s disease. Among them, 102 participants improved the cognitive domain assessment scale, and the results showed that several EEG indexes were correlated with cognitive domain function score. The blood samples of 105 normal and MCI participants were tested for blood P-tau217 concentration. The relative power of right temporal lobe and right parietal lobe was negatively correlated with blood P-tau217 concentration, and the relative power of right parietal lobe was an independent influencing factor for the increase of blood P-tau217 level (AUC = 0.68).

**Conclusion:**

Quantitative EEG indicators show potential as a complementary screening approach for AD-related cognitive impairment. Further validation in prospective, multicenter cohorts is needed before implementation in clinical practice.

## 1 Introduction

Alzheimer’s disease (AD), a prevalent neurodegenerative disorder in the elderly, is the most common cause of late-onset dementia, resulting in a considerable societal burden (1). While dementia can result from multiple etiologies, AD pathology accounts for a substantial proportion of cases. The advanced stage of AD is characterized by profound cognitive deficits, loss of autonomy in daily living, and significant psychiatric symptoms, leading to substantially reduced quality of life (2). Prior to the stage of dementia, individuals experience a period of mild cognitive impairment (MCI), presenting with decreased cognitive performance that affects complex daily activities to a minor extent. Epidemiological studies reveal a 12.2% prevalence of MCI among Chinese residents aged 55 years and older (1), with 40–75% of these cases attributable to AD (3). Annually, 10–15% of MCI cases progress to AD dementia (4). With the aging of society, early screening of AD-related cognitive impairment using a convenient and easily generalizable method has become increasingly important (5, 6). Electroencephalography (EEG), as a non-invasive, cost-effective tool, offers quantitative data on synaptic function with millisecond-level temporal resolution (7). Evidence shows that synaptic injury and loss are characteristic of AD neurodegeneration and occur before structural atrophy (8, 9). Thus, EEG may be a suitable candidate for early screening of AD-related cognitive impairment.

Previous studies have shown that quantitative EEG (qEEG) indicators can effectively distinguish various stages of AD-related cognitive impairment. In AD dementia and MCI patients, resting-state EEG with eyes closed reveals increased delta (0.5–4 Hz) and theta (4–8 Hz) power with decreased alpha (8–13 Hz) and beta (13–30 Hz) power. Additionally, the dominant alpha rhythm, which is typically maximal over occipital regions in healthy older adults, shows an anterior shift (increased frontal alpha power) in patients with AD-related cognitive impairment. The degree of EEG changes in MCI patients is intermediate between AD dementia and healthy controls (7, 10–13). Using individual EEG metrics such as theta frequency band absolute power/relative power and alpha2 cortical source activity, the area under the curve (AUC) for differentiating AD dementia from healthy controls ranges from 0.69 to 0.91 (13, 14), and from 0.60 to 0.75 for MCI versus healthy controls (13, 15). EEG composite indicators, such as frequency band ratios, improve disease classification rates over single-band indicators. Schmidt et al. achieved an AUC of 0.92 using the alpha/theta power ratio to distinguish AD dementia from healthy controls (16). Recently, qEEG analyses were advanced by utilizing machine learning (ML) algorithms. Multiple studies reported classification rates around 0.80–0.90 when distinguishing between MCI/AD and normal groups (17–20); however, these studies were limited by relatively small sample sizes (22–65 MCI cases). In 2023, Jiao et al. investigated qEEG data from 330 AD dementia patients, 189 MCI patients, and 246 healthy controls using ML techniques, achieving classification accuracy exceeding 70% (21).

Neuropsychological scales, encompassing cognitive screening and domain-specific assessment tools, are essential for evaluating cognitive dysfunction. Prior studies have revealed substantial correlations between EEG indicators and Mini-Mental State Examination (MMSE) scores (10, 13, 14). While domain-specific cognitive tests are invaluable for diagnosing MCI and determining the causes of cognitive impairment, their administration and interpretation are intricate and time-intensive, with high requirements for both participants and examiners. Moreover, the MMSE has recognized limitations, including poor sensitivity to subtle cognitive changes and ceiling effects in highly educated individuals. Thus, it is virtually infeasible to comprehensively implement these tests in primary healthcare facilities. Utilizing EEG indicators to predict domain-specific cognitive impairments might potentially substitute for extensive scale assessments and improve diagnostic accuracy and early screening efficiency for AD. Several prior studies have reported correlations between EEG indicators and memory scores (22–24); however, comprehensive investigations into the relationships between various EEG indicators and domain-specific cognitive test scores, as well as their predictive capacity, remain sparse.

AD biomarkers are vital for diagnosis and screening. However, their practical applications are limited by invasive procedures, high costs, and/or the involvement of radioactive substances. Consequently, research has shifted toward examining the correlation between EEG indicators and AD biomarkers, aiming to identify potential substitutes. Such research has mostly focused on cerebrospinal fluid (CSF) biomarkers, revealing multiple EEG indicators related to CSF Aβ42 and P-tau levels in MCI patients (25, 26). Research exploring the connection between qEEG and AD plasma biomarkers remains sparse (27). A review of previous literature uncovered a solitary study by Gonzalez-Escamilla et al., which examined the relationship between brain functional connectivity and blood Aβ levels in MCI and healthy individuals, revealing a negative correlation between hemispheric functional connectivity and blood Aβ concentration in healthy participants (28).

Thus, this study aimed to assess the utility of qEEG in the early screening of AD-related cognitive impairment. EEG markers to distinguish severity levels of cognitive impairment, to predict patterns of domain-specific cognitive injury, and to predict AD plasma biomarkers were screened and evaluated respectively, merging traditional statistical methods with ML algorithms.

## 2 Methods

### 2.1 Study population

Participants were recruited from the neurology inpatient and memory clinics at Sichuan Provincial People’s Hospital consecutively. Eligibility required Chinese citizenship, age 55 years or older, fluency in Chinese, and consent to participate. Exclusion criteria encompassed: (1) conditions significantly affecting life expectancy; (2) neurological disorders other than AD that cause cognitive impairment; (3) severe mental illness; (4) inability to complete the study. The Ethics Committee of Sichuan Academy of Medical Sciences and Sichuan Provincial People’s Hospital approved this study, and all participants provided written informed consent prior to enrollment.

Participants underwent thorough information collection and clinical assessment encompassing demographic information (age, sex, education level), medical history, and a battery of blood tests including liver and kidney function, thyroid function, syphilis antibody, HIV antibody, folic acid, vitamin B_12_ levels, and brain magnetic resonance imaging (MRI)/computed tomography (CT). Cognitive evaluations and EEG were also administered.

Based on established clinical diagnostic criteria (Petersen and Morris for MCI (29); McKhann et al. for AD dementia (30)), participants were classified into three groups: normal cognition, MCI, and AD dementia. Of note, diagnoses were established based on clinical assessment without confirmatory CSF or positron emission tomography (PET) biomarkers; therefore, the MCI and AD dementia groups represent clinically defined AD-related cognitive impairment rather than biomarker-confirmed AD pathology. The proportion of non-AD etiologies within the MCI group was not systematically assessed, which is acknowledged as a limitation.

### 2.2 Neuropsychological assessment

Each participant underwent a comprehensive battery of neuropsychological assessments, including the MMSE (31), Montreal Cognitive Assessment (MoCA) (32), Auditory Verbal Learning Test-Huashan version (AVLT) (33), Common Objects Memory Test (COMT) (34), Shape-Trail Making Test (STT) (35), Rey-Osterrieth Complex Figure Test (ROCF) (36), Digit Span Test (DST) (37), Boston Naming Test (BNT) (38), and Animal Fluency Test (AFT) (39). These tests, translated and adapted from Western sources to align with Chinese culture, were validated in the Chinese population. MMSE and MoCA assessed overall cognition, and the remaining tests assessed five cognitive domains: memory, attention, visuospatial function, executive function, and language function. Raw scores from domain-specific cognitive tests were z-normalized using the mean and standard deviation (SD) of localized normative data for each test individually, with age or education adjustment. The formula used was *Z* = (raw score – mean)/SD. This normalization was applied to facilitate comparisons across tests with different scoring ranges.

### 2.3 Blood sample collection and treatment

Venous blood was collected using EDTA vacuum tubes, refrigerated at 4 °C, and centrifuged at 2000 rpm for 10 min within 2 h. Serum aliquots of 500 μL were transferred to 1.0 mL polypropylene tubes and stored at −80 °C. Plasma phosphorylated tau protein 217 (P-tau217) concentrations were analyzed by single molecule array (reagent batch number: 20230829, AstraBio, Suzhou, China) assay using the fully automated instrument AST-DX90 Analyzer (AstraBio, Suzhou, China) following the manufacturer’s instructions, which is suitable for detecting ultra-low abundance proteins in peripheral blood (40).

### 2.4 EEG data collection and preprocessing

**Figure 1:**
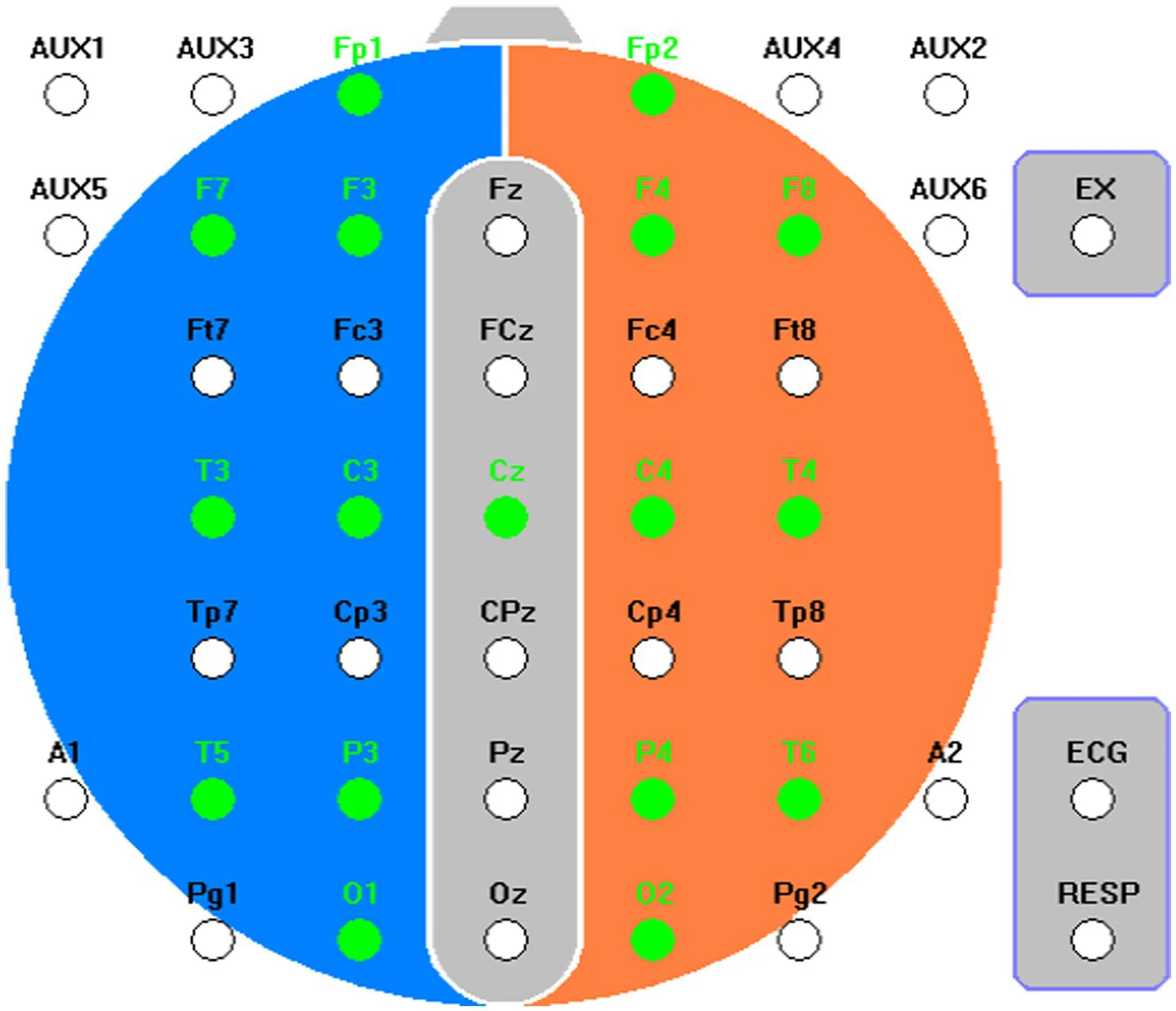
The locations of brain electrodes adopted in the present study (marked green).

Participants underwent a 15-min EEG recording session in a quiet room between 14:00 and 17:30 using a digitized EEG machine (SYMTOP Instrument Co., Beijing, China) which utilized a clinical standard electrode montage adhering to the International 10–20 system (41). The configuration of the present study comprised 18 channels with earlobes A1 and A2 as references and 16 Ag-AgCl electrodes positioned at Fp1, Fp2, F3, F4, C3, C4, P3, P4, O1, O2, F7, F8, T3, T4, T5, and T6, grounded at Fpz and referenced at FCz (Figure 1). Additional earlobe electrodes were included for re-referencing. The sampling rate was 200 Hz, and impedance was kept below 10 kΩ.

EEG recordings began with a 2-min eyes-closed resting-state condition. Participants were instructed to relax with eyes closed while remaining awake, followed by cycles of eyes opening and closing, and to minimize unnecessary movements. Vigilance was continuously monitored by a trained technician who observed participants through a one-way window; any signs of drowsiness (e.g., slow eye movements, decreased alpha activity) prompted verbal re-alerting.

Raw EEG data were preprocessed in Python using the open-source MNE library. Channels with excessive noise or flat signals were identified via visual inspection and removed (average 1.2 channels per participant). A bandpass filter (0.5–40 Hz) was applied to match the subsequent spectral analysis range. Data were re-referenced to the average of A1 and A2. Continuous EEG was segmented into 2-s epochs. Independent component analysis (ICA) was applied to remove components corresponding to eye blinks, horizontal eye movements, and cardiac artifacts. Epochs with amplitude exceeding ±100 μV were automatically rejected. On average, 8.3% of epochs were rejected, and all participants had at least 80 artifact-free epochs. No participant was excluded entirely due to poor data quality.

### 2.5 EEG data analysis and feature extraction

This study utilized Hann window fast Fourier transform spectrum analysis to compute power spectral densities (PSDs) of EEG signals across various scalp regions. PSDs were computed using Welch’s method with 2-s Hanning windows, 50% overlap, and a frequency resolution of 0.5 Hz. Previous research indicates that low-frequency alpha1 wave (8–10.5 Hz) reflects decreased levels of general brain arousal, preparation, and attention, while high-frequency alpha2 (10.5–13 Hz) and low-frequency beta1 waves (13–20 Hz) are linked to diminished perception, somatic movement, and memory. In contrast, high-frequency beta2 (20–30 Hz) and gamma (30–40 Hz) waves primarily relate to sensorimotor and cognitive processes (42). Thus, this study segmented the 0.5–40 Hz EEG data into seven frequency bands: delta (0.5–4 Hz), theta (4–8 Hz), alpha1 (8–10.5 Hz), alpha2 (10.5–13 Hz), beta1 (13–20 Hz), beta2 (20–30 Hz), and gamma (30–40 Hz). The average number of epochs recorded with eyes closed was 112.5 (±4.95), with no significant differences across diagnostic groups.

EEG indicators, such as absolute power, relative power, total power, and power ratios, were calculated. Total power was defined as the cumulative power across all frequency bands within the 0.5–40 Hz range. Absolute power (AP) referred to the power within each specific frequency band, while relative power (RP) was calculated as the percentage of each frequency band’s absolute power relative to the total power. Absolute power for each frequency band was calculated as the average power across epochs for each channel and region, followed by log transformation: log10 (absolute power) to obtain decibels (dB). Relative power was calculated as the percentage of absolute power within a frequency band relative to total power (0.5–40 Hz) per region, also log-transformed.

This study also incorporated power ratios—defined as the ratio of absolute power between two frequency bands—as EEG indicators, including alpha2/alpha1, alpha1/theta, alpha1/delta, delta/theta, and theta/beta1. Sixteen electrodes were categorized into ten scalp regions: left frontal (Fp1, F3, F7), right frontal (Fp2, F4, F8), left central (C3), right central (C4), left parietal (P3), right parietal (P4), left temporal (T3, T5), right temporal (T4, T6), left occipital (O1), and right occipital (O2). This resulted in 200 EEG indicators, including 70 absolute power values (7 frequency bands × 10 scalp regions), 70 relative power values (7 frequency bands × 10 scalp regions), 10 total power values (each total power × 10 scalp regions), and 50 power ratio indicators (5 power ratio indicators × 10 scalp regions). To achieve an approximate normal distribution for further analysis, the original values of absolute power, total power, relative power, and power ratios were logarithmically transformed to base-10 dB.

### 2.6 Statistical methods

Data analysis was conducted using SPSS 26.0 software and Python (for ML analyses). A two-sided test with *α* = 0.05 was applied, where *p* < 0.05 indicated statistical significance. Measurement data were expressed as mean ± standard deviation, and count data as frequency (percentage). Normality was assessed using the Shapiro–Wilk test; parametric tests (one-way analysis of variance [ANOVA] or independent sample *t*-tests) were used for normally distributed data, and non-parametric alternatives (Kruskal–Wallis or Mann–Whitney *U*) otherwise. Count data were examined using the chi-square test. Age, sex, and education were included as covariates in all ANOVA and regression models.

Given the large number of EEG indicators (200 features) and multiple comparisons across groups and cognitive domains, the Benjamini–Hochberg false discovery rate (FDR) method was applied to control for type I error. A corrected significance threshold of *q* < 0.05 was used for all correlation analyses and group comparisons involving multiple EEG features. Results that remained significant after FDR correction are indicated in the tables. Findings that did not survive FDR correction are reported as exploratory and are explicitly labeled as such.

Spearman’s bivariate correlation analysis was adopted to evaluate the correlation between continuous data from two groups when assumptions of normality were not met. ANOVA with repeated measures was utilized to assess differences in EEG indicators across various scalp regions among multiple groups. Bonferroni correction was used for simple effects analysis. The receiver operating characteristic (ROC) curve assessed the discriminatory power of EEG indicators for group classification, derived from logistic regression models.

### 2.7 Machine learning

Utilizing the XGBoost algorithm, this study analyzed EEG features and demographic data from the three groups: AD dementia, MCI, and healthy controls. XGBoost, an advanced gradient-boosted decision tree, iteratively improves classification accuracy by minimizing the gradient discrepancy between predicted and actual outcomes. Regularization techniques were applied to reduce overfitting, optimize the computational tree, and ensure swift, precise predictions (42). To avoid overfitting and ensure robust performance estimation, we employed nested cross-validation. The dataset was stratified by diagnosis and split into 5 outer folds for training/testing, with an inner 5-fold cross-validation within each training fold for hyperparameter tuning using Bayesian optimization. Hyperparameters tuned included learning rate, maximum tree depth, subsample ratio, and column sampling ratio. This procedure was repeated 100 times with different random seeds to obtain stable performance estimates. Due to class imbalance (AD dementia: 154, MCI: 116, controls: 90), we report balanced accuracy in addition to standard accuracy. Classification performance was quantified by the AUC of the ROC curve. Feature importance was extracted using the built-in gain-based importance metric in XGBoost. Demographic variables (age, sex, education) were initially included; a sensitivity analysis excluding these variables was performed to assess their contribution to classification performance.

## 3 Results

### 3.1 General information of participants

A total of 360 participants were recruited from the Department of Neurology at Sichuan Academy of Medical Sciences and Sichuan Provincial People’s Hospital, comprising 154 AD dementia patients, 116 MCI patients, and 90 healthy controls. General clinical characteristics are presented in Table 1. No significant differences were noted in sex or common comorbidities among the three groups. However, age, educational level, MMSE, and MoCA scores exhibited significant differences (*p* < 0.05).

**Table 1:** Clinical profiles of the three groups at baseline.

Characteristics	NC	MCI	AD	*p* value
N. of each group	90	116	154	
N. of male (%)	34 (37.78%)	51 (43.97%)	56 (36.36%)	>0.05
Age (y)	66.13 ± 10.43	69.94 ± 8.82	74.70 ± 8.80	<0.05
Education years (y)	10.43 ± 4.42	8.84 ± 4.11	8.07 ± 4.75	<0.05
N. with HLP (%)	28 (35.00%)	39 (39.00%)	67 (50.00%)	>0.05
N. with stroke (%)	7 (7.78%)	11 (6.09%)	15 (9.87%)	>0.05
N. with HTN (%)	38 (42.70%)	53 (46.09%)	61 (40.13%)	>0.05
N. with DM (%)	16 (17.78%)	31 (27.19%)	31 (20.13%)	>0.05
N. with anxiety (%)	48 (53.33%)	71 (61.21%)	79 (51.63%)	>0.05
MMSE score	28.14 ± 1.86	26.06 ± 2.44	16.03 ± 5.99	<0.05
MoCA score	23.52 ± 4.77	18.50 ± 3.45	10.58 ± 4.74	<0.05

N., number; NC, normal control; MCI, mild cognitve impiarment; AD, Alzheimer’s disease dementia; HLP, hyperlipidaemia; HPN, hypertention; DM, diabetes mellitus; MMSE, mini-mental state examination; MoCA, Montreal Cognitive Assessments.

### 3.2 Classification of AD-related cognitive impairment based on quantitative EEG data

**Figure 2:**
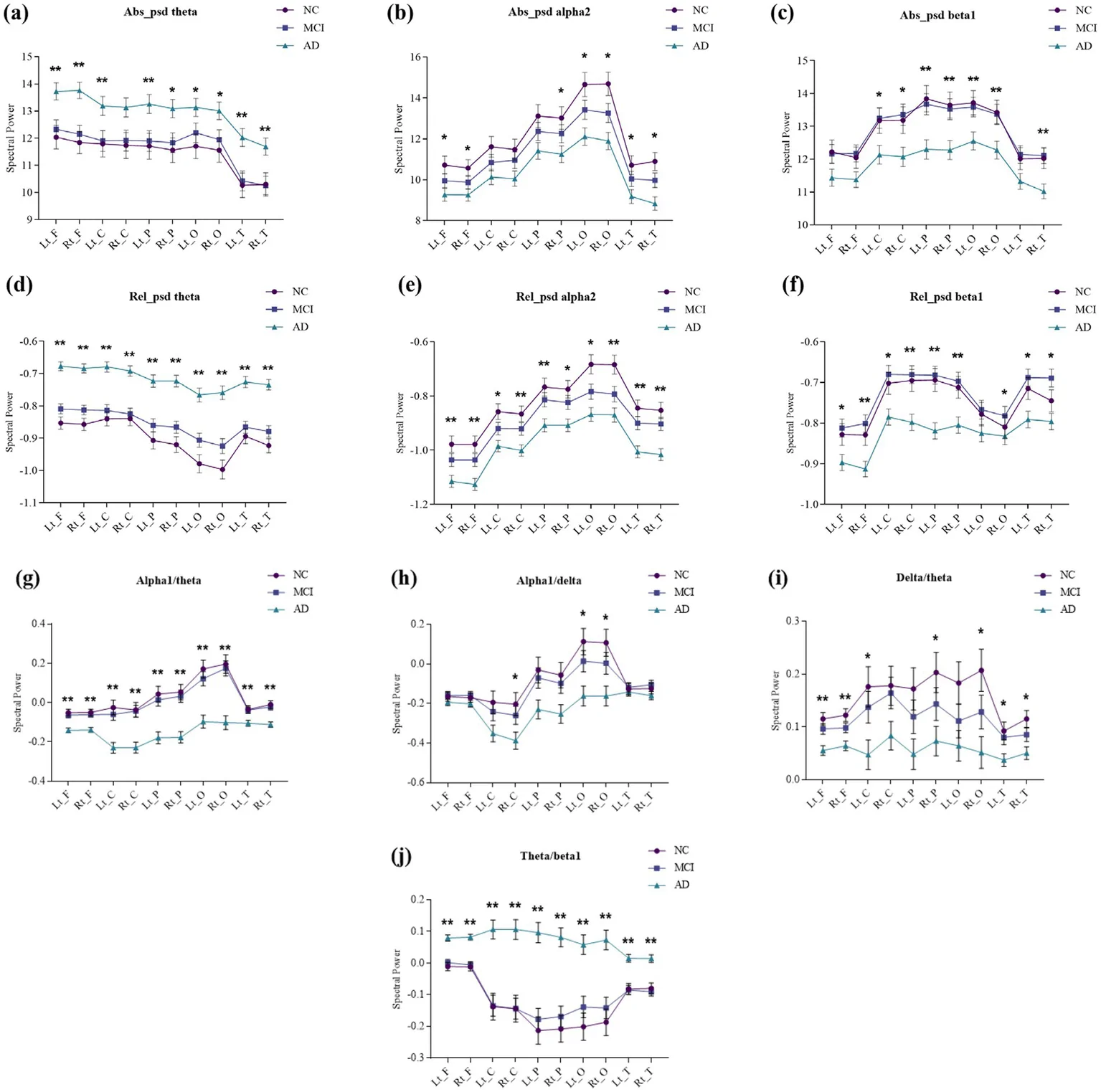
Comparisons of EEG power spectrums among the three groups of participants. The horizontal coordinates represented ten brain regions, and the vertical coordinates represented the estimated marginal mean values of power spectral density. ^*^ indicates that the power spectral density in the brain area was statistically different between AD dementia and normal groups or between AD dementia and MCI groups. ^**^ indicates that the power spectral density in the brain area was statistically different in both of the comparisons. Lt_F, left frontal lobe; Rt_F, right frontal lobe; Lt_C, left center; Rt_C, right center; Lt_P, left parietal lobe; Rt_P, right parietal lobe; Lt_O, left occipital lobe; Rt_O, right occipital lobe; Lt_T, left temporal lobe; Rt_T, right temporal lobe. NC,cognitive normal group;MCI, mild cognitive impairment group;AD,Alzheimer’s disease dementia group.

**Figure 3:**
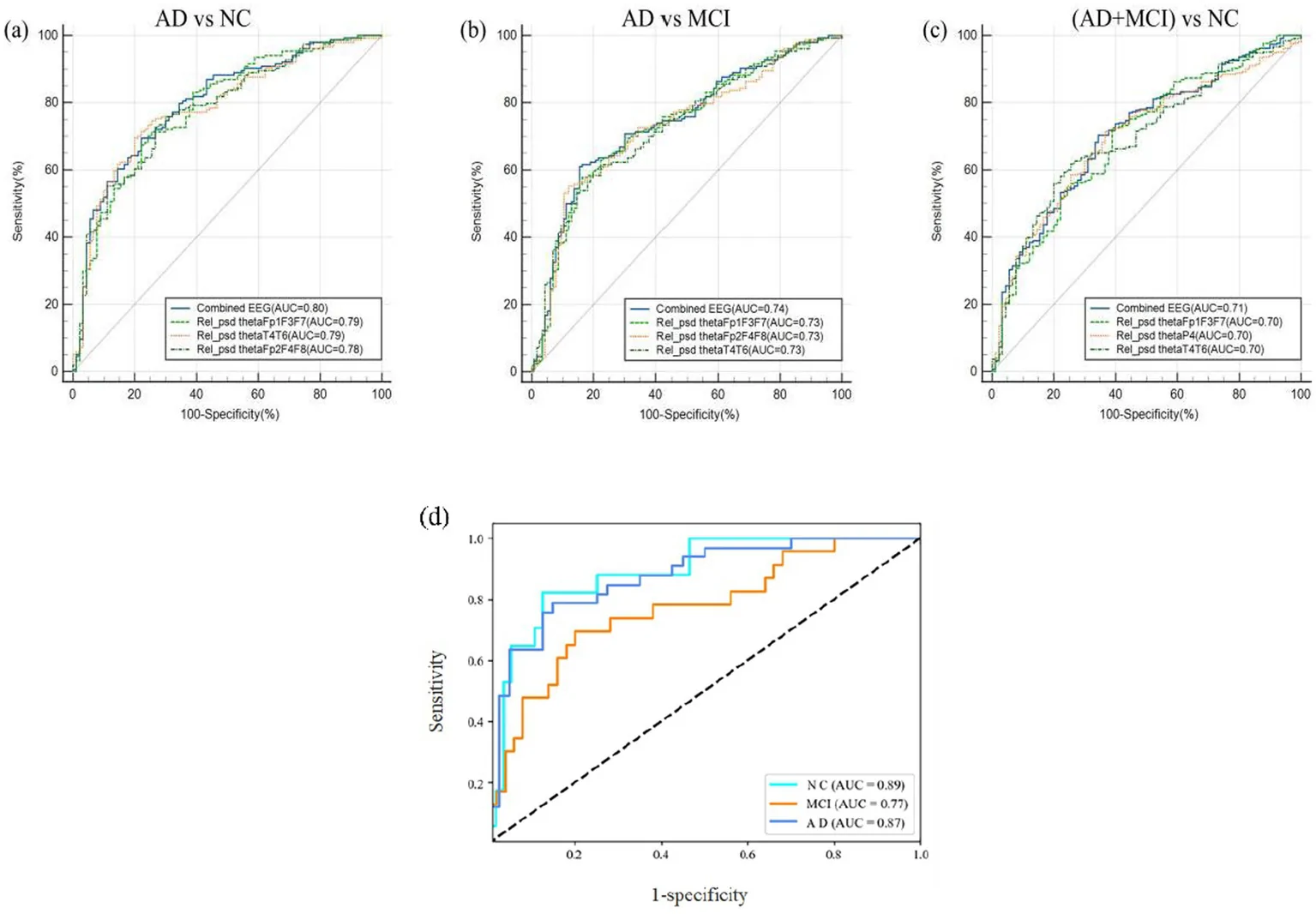
**(a–c)** ROC curves of the optimal EEG indicators in distinguishing the three groups using conventional statistical analysis. **(a)** AD vs. NC; **(b)** AD vs. MCI; **(c)** (AD+MCI) vs. NC; **(d)** ROC curves of the optimal classification model of the three groups using machine learning analysis.

A total of 82 EEG indicators were identified with significant differences in at least one comparison among the three groups (i.e., AD dementia vs. normal, MCI vs. normal, AD dementia vs. MCI), including 10 types of EEG power spectra: AP in theta, alpha2, beta1; RP in theta, alpha2, beta1; and power ratios of alpha1/theta, alpha1/delta, delta/theta, and theta/beta1 (Figure 2). Notably, 54 EEG indicators showed significant differences in two comparisons (i.e., AD dementia vs. normal; AD dementia vs. MCI). AD dementia and MCI groups were combined as a cognitive impairment group for further analysis. Thus, these 54 indicators underwent ROC curve analysis to assess the ability to classify cognitive impairments in three comparisons: AD dementia versus normal, AD dementia versus MCI, and cognitive impairment (AD dementia + MCI) versus normal. The AUC values for distinguishing AD dementia from normal ranged from 0.57 to 0.79; for differentiating AD dementia from MCI, the AUC ranged from 0.57 to 0.73; and for identifying cognitive impairment from normal, the AUC ranged from 0.57 to 0.70. As shown in Table 2 and Figures 3a–c, based on the logistic regression model, for AD dementia vs. normal, the top three EEG indexes are left frontal lobe theta relative power, right temporal lobe theta relative power and right frontal lobe theta relative power, with AUC ranging from 0.78 to 0.79. These three EEG indexes are combined as a joint index, and the joint index AUC is 0.80. For AD dementia vs. MCI, the top three EEG indexes are also left frontal lobe theta relative power, right frontal lobe theta relative power and right temporal lobe theta relative power, with an AUC of about 0.73. These three EEG indexes are combined as a joint index with an AUC of 0.74. For cognitive impairment (AD dementia +MCI) vs. normal, the top three EEG indexes are the relative power of right parietal lobe, right temporal lobe and left frontal lobe, and the AUC is about 0.70. The joint index AUC is 0.71.

**Table 2:** EEG indicators with the highest AUC values in identifying AD related cognitive impairment via ROC curve analysis.

EEG indicators	Sensibility	Specificity	Cut-offs	AUC	*p* value
AD vs. NC					
Left frontal theta relative power	0.71	0.74	−0.77	0.79	<0.05
Right temporal theta relative power	0.69	0.80	−0.83	0.79	<0.05
Right frontal theta relative power	0.73	0.73	−0.77	0.78	<0.05
Combined indicator 1	0.69	0.78	0.64	0.80	<0.05
AD vs. MCI					
Left frontal theta relative power	0.58	0.84	−0.70	0.73	<0.05
Right frontal theta relative power	0.55	0.88	−0.71	0.73	<0.05
Right temporal theta relative power	0.58	0.81	−0.76	0.73	<0.05
Combined indicator 1	0.61	0.84	0.61	0.74	<0.05
(AD+MCI) vs. NC					
Right parietal theta relative power	0.71	0.63	−0.89	0.70	<0.05
Right temporal theta relative power	0.62	0.74	−0.85	0.70	<0.05
Left frontal theta relative power	0.73	0.61	−0.83	0.70	<0.05
Combined indicator 2	0.70	0.66	0.73	0.71	<0.05

NC, normal control; MCI, mild cognitve impiarment; AD, Alzheimer’s disease dementia; ROC, Receiver Operating Characteristic Curve; AUC, Area Under Curve.

This study employed ML techniques (XGBoost algorithm) to further mine the data. The model utilized 200 EEG indicators and demographic data (sex, age, education level) as input features. Figure 3d illustrates the classification performance. For three-level classification, the highest balanced accuracy was 0.71, with a mean balanced accuracy of 0.52 ± 0.05. For classification of AD dementia, the AUC range is 0.70–0.93, the highest AUC was 0.87, with a mean AUC of 0.78 ± 0.05. When classifying the MCI group, the AUC range is 0.47–0.78, the highest AUC was 0.77, with a mean AUC of 0.64 ± 0.07. For the normal control group, the AUC range is 0.54–0.89, the highest AUC was 0.89, with a mean AUC of 0.71 ± 0.07. Feature importance analysis revealed that theta relative power in frontal regions and age were among the top contributors to model performance. A sensitivity analysis excluding demographic variables yielded slightly lower but consistent AUC patterns.

### 3.3 Identification of EEG markers for domain-specific cognitive impairment

A subset of 102 participants completed the full battery of domain-specific cognitive assessments; this subset was derived from consecutive enrollment after the domain-specific protocol was introduced, with no additional selection criteria. These participants completed assessments across multiple cognitive domains, including verbal (AVLT (33)) and non-verbal (COMT (34), ROCF-recall (36)) episodic memory, attention (DST (37), STT-A (35)), executive function (STT-B (35)), visuospatial function (ROCF-copy (36)), and language function (BNT (38) and AFT (39)). Table 3 illustrates that while sex and education levels showed no significant differences across groups, age, *Z* values of key measures of domain-specific cognitive tests, MMSE, and MoCA scores displayed significant variations among the three groups.

**Table 3:** Neuropsychological profiles of participants who completed a comprehensive battery of neurocognitive assessments.

Characteristics	NC	MCI	AD	*p* value
N.	38	42	22	
N. of male (%)	14 (36.84%)	19 (45.24%)	13 (59.09%)	>0.05
Age (y)	65.53 ± 9.11	69.36 ± 9.62	71.91 ± 7.17	<0.05
Education years (y)	12.46 ± 3.02	11.37 ± 2.75	10.64 ± 4.08	>0.05
DST forward (*Z* score)	−0.16 ± 1.02	−0.91 ± 1.10	−0.86 ± 1.22	<0.01
DST backward (*Z* score)	−0.18 ± 1.39	−0.94 ± 0.87	−1.06 ± 1.24	<0.01
AVLT 5-min recall (*Z* score)	1.11 ± 0.89	−0.62 ± 1.04	−2.32 ± 0.58	<0.01
AVLT 30-min recall (*Z* score)	1.04 ± 1.08	−0.85 ± 1.20	−2.43 ± 0.47	<0.01
AVLT recognition (*Z* score)	0.81 ± 0.52	−0.27 ± 1.32	−2.74 ± 1.48	<0.01
COMT 5-min recall (*Z* score)	0.21 ± 0.32	−0.07 ± 0.85	−2.56 ± 0.29	<0.01
COMT 30-min recall (*Z* score)	0.60 ± 0.70	0.15 ± 0.75	−3.23 ± 1.07	<0.01
COMT recognition (*Z* score)	0.37 ± 0.04	0.39 ± 0.03	−4.87 ± 2.73	<0.01
STT-A (*Z* score)	0.14 ± 1.03	−0.93 ± 0.99	−2.50 ± 2.20	<0.01
STT-B (*Z* score)	0.46 ± 0.76	−0.81 ± 0.98	−2.62 ± 1.81	<0.01
Rey copy (*Z* score)	−0.27 ± 0.68	−1.32 ± 1.79	−2.36 ± 2.45	<0.01
Rey recall (*Z* score)	0.13 ± 1.01	−1.24 ± 0.92	−2.12 ± 0.69	<0.01
BNT (*Z* score)	−0.89 ± 0.61	−1.61 ± 0.78	−2.40 ± 1.09	<0.01
AFT (*Z* score)	0.72 ± 1.10	−0.92 ± 1.03	−1.55 ± 1.08	<0.01
MMSE	28.82 ± 1.28	27.32 ± 1.62	21.05 ± 5.19	<0.01
MoCA	24.63 ± 5.30	20.44 ± 2.94	14.79 ± 4.35	<0.01

N., number; NC, normal control; MCI, mild cognitive impiarment; AD, Alzheimer’s disease dementia; AVLT, Auditory Verbal Learning Test-Huashan version; COMT, Common Objects Memory Test; STT, Shape-Trail Making Test; ROCF, Rey-Osterrieth Complex Figure Test; DST, Digit Span Test; BNT, Boston Naming Test; AFT, Animal Fluency Test; MMSE, mini-mental state examination; MoCA, Montreal Cognitive Assessments.

Spearman correlation analysis was conducted on 200 quantitative EEG indicators and 16 cognitive indicators encompassing the MMSE, MoCA total score, and 14 key measures of domain-specific cognitive tests. After FDR correction (*q* < 0.05), 255 correlations reaching statistical significance (Table 4).

**Table 4:** Results of correlation analysis between neurocognitive scores and EEG indicators.

Neurocognitive scores	N. of EEG indicators with significant CI	EEG indicator with the highest |CI|	CI with the highest absolute value	*p* value
MMSE	21	Left occipital beta1 AP	0.35	<0.01
MoCA	4	Left occipital beta1 AP	0.29	<0.01
DST forward (*Z* score)	2	Right frontal delta AP	−0.23	<0.05
DST backward (*Z* score)	1	Left temperal delta AP	−0.20	<0.05
AVLT 5-min recall (*Z* score)	18	Left occipital alpha2 RP	0.28	<0.01
AVLT 30-min recall (*Z* score)	21	Left occipital beta1 RP	0.29	<0.01
AVLT recognition (*Z* score)	7	Left occipital beta1 RP	0.26	<0.05
COMT 5-min recall (*Z* score)	18	Right parietal alpha2 RP	0.62	<0.05
COMT 30-min recall (*Z* score)	7	Left temporal theta RP	−0.56	<0.05
COMT recognition (*Z* score)	30	Left frontal theta/beta1	−0.82	<0.01
STT-A (*Z* score)	9	Right occipital theta RP	−0.24	<0.05
STT-B (*Z* score)	23	Left occipital theta/beta1	−0.26	<0.01
Rey copy (*Z* score)	18	Left temporal beta1 AP	0.28	<0.01
Rey recall (*Z* score)	53	Left temporal alpha2 RP	0.31	<0.05
		Right central alpha2 RP	0.30	<0.01
AFT (*Z* score)	7	Right temporal delta/theta	0.27	<0.01
BNT (*Z* score)	16	Left frontal alpha2 AP	0.27	<0.01

N., number; AVLT, Auditory Verbal Learning Test-Huashan version; COMT, Common Objects Memory Test; STT, Shape-Trail Making Test; ROCF, Rey-Osterrieth Complex Figure Test; DST, Digit Span Test; BNT, Boston Naming Test; AFT, Animal Fluency Test; MMSE, mini-mental state examination; MoCA, Montreal Cognitive Assessments; AP, Absolute power; RP, Relative power; CI, correlation index.

Then, the participants were divided into two groups based on the *Z* values of key measures of domain-specific cognitive tests (43): those with impaired cognitive domain function and those without. A *Z* value for AVLT short-delay recall, long-delay recall, or recognition ≤ − 1.5 indicated impaired verbal episodic memory; otherwise, verbal episodic memory was considered unimpaired. Similarly, a *Z* value for Rey recall, COMT short-delay recall, COMT long-delay recall, or COMT recognition ≤−1.5 signified impaired non-verbal episodic memory; otherwise, non-verbal episodic memory was deemed unimpaired. The episodic memory impairment group included individuals with deficits in verbal, non-verbal, or both types of episodic memory. A similar classification method was implemented for executive function, visuospatial function, language function, and attention function. ANOVA with repeated measures was adopted for analysis, with age, sex, and education as covariates. Significant differences were found in multiple EEG indicators between impaired and non-impaired groups in episodic memory, executive function, and visuospatial function. ROC curve analysis was used to evaluate the predictive accuracy of these EEG indicators for domain-specific cognitive impairment. Table 5 lists the top three EEG indicators with the highest AUC for each cognitive domain after FDR correction.

**Table 5:** EEG indicators with the highest AUC for identifying the impairment of each cognitive domain.

EEG indicators	Sensitivity	Specificity	Cut-offs	AUC	*p* value
Episodic memory					
Left occipital beta1 RP	0.66	0.62	−0.77	0.64	<0.05
Left occipital theta/beta1	0.70	0.58	−0.23	0.62	<0.05
Non-verbal episodic memory					
Right occipital theta RP	0.63	0.70	−0.87	0.66	<0.05
Right frontal alpha2 RP	0.79	0.66	−1.07	0.70	<0.05
Right central alpha2 RP	0.76	0.66	−0.94	0.70	<0.05
Right temporal beta1 RP	0.71	0.53	−0.71	0.63	<0.05
Left frontal alpha1/theta	0.76	0.48	−0.04	0.63	<0.05
Right central alpha1/delta	0.79	0.53	−0.23	0.68	<0.05
Left occipital theta/beta1	0.76	0.56	−0.23	0.67	<0.05
Executive function					
Right central theta AP	0.76	0.58	12.62	0.65	0.027
Left occipital theta AP	0.68	0.64	12.45	0.65	<0.05
Left occipital theta RP	0.64	0.75	−0.82	0.69	<0.05
Left occipital theta/beta1	0.52	0.82	0.03	0.69	<0.05
Visuospatial function					
Right occipital alpha2 AP	0.52	0.88	9.96	0.72	<0.05
Left temporal beta1 AP	0.96	0.41	13.06	0.73	<0.05
Right parietal theta RP	0.88	0.52	−0.89	0.68	<0.05
Left temporal alpha2 RP	0.56	0.80	−1.06	0.72	<0.05
Left parietal beta1 RP	0.56	0.77	−0.81	0.68	<0.05
Left temporal alpha2/alpha1	0.42	0.91	−0.09	0.67	<0.05
Right central alpha1/delta	0.52	0.81	−0.62	0.68	<0.05
Left occipital alpha1/delta	0.72	0.63	−0.12	0.68	<0.05
Right parietal theta/beta1	0.52	0.80	0.04	0.70	<0.05

AUC, Area Under Curve; AP, Absolute power; RP, Relative power.

### 3.4 Identification of EEG markers for elevated plasma P-tau217 concentration

In 105 non-dementia participants, plasma P-tau217 levels were analyzed against 200 EEG indicators. The results indicated a significant negative correlation between plasma P-tau217 and the RP of beta1 in the right temporal area (*r* = −0.21, *p* < 0.05), along with a negative correlation with the RP of beta2 in the right parietal area (*r* = −0.19, *p* < 0.05), both surviving FDR correction.

Then, the 105 non-dementia participants were categorized into three groups according to the tertiles of their plasma P-tau217 levels (*n* = 35 in each group). The top third represented the high P-tau217 group, while the bottom third represented the low P-tau217 group. The results showed that the average concentration in the low group was 4.69 ± 1.82 pg/mL, whereas the high group averaged 27.67 ± 18.00 pg/mL. No significant differences in sex, age, educational level, or comorbidities (stroke, hypertension, hyperlipidemia, anxiety, and depression) were found between the low and high P-tau217 groups; however, a significant difference was observed in diabetes history (*p* < 0.05). EEG indexes (delta relative power of right frontal lobe, beta1 relative power of right parietal lobe, beta2 relative power of right parietal lobe), diabetes history and factors (gender, age, years of education, MMSE, MOCA) that may have an influence on blood P-tau217 level were substituted into the binary logistic regression equation. Table 6 indicated that only the right-parietal beta1 RP significantly predicted blood P-tau217 level in the top tertile (OR = 0.007, 95% CI 0.000–0.708, *p* < 0.05) via binary logistic regression (AUC = 0.68).

**Table 6:** Identifying EEG indicator which best predicted the elevation of blood P-tau217 via logistic regression analysis.

Characteristics	*B* value	SE	OR (95% CIs)	*p* value
Right frontal delta RP	−2.485	1.510	0.083 (0.004–1.607)	0.100
Right parietal beta1 RP	−4.916	2.332	0.007 (0.000–0.708)	0.035
Right parietal beta2 RP	0.309	1.499	0.363 (0.072–25.698)	0.836
DM	−1.411	0.730	0.244 (0.058–1.019)	0.053
Gender	0.799	0.641	2.222 (0.633–7.806)	0.213
Age	0.010	0.033	1.011 (0.947–1.078)	0.751
Education level	−0.082	0.089	0.921 (0.774–1.096)	0.354
MMSE	0.252	0.157	1.287 (0.946–1.750)	0.108
MoCA	−0.075	0.074	0.928 (0.803–1.072)	0.310

OR, odds ratio; DM, diabetes mellitus; RP, Relative power; MMSE, mini-mental state examination; MoCA, Montreal Cognitive Assessments; CI, confidence interval; SE, standard error.

## 4 Discussion

In this study, 360 participants were included, among which 102 participants completed the complete cognitive domain assessment scale, and 105 normal and MCI participants’ blood samples were tested for blood P-tau217 concentration. According to the established clinical diagnostic criteria, the participants were divided into three groups: cognitive normal group, mild cognitive impairment group (MCI) and dementia group with Alzheimer’s disease. By analyzing 200 EEG indexes from three main dimensions, the application potential of quantitative EEG (qEEG) in early screening and diagnosis of cognitive impairment related to Alzheimer’s disease was discussed. The results indicated that qEEG provides valuable and comprehensive data for screening AD-related cognitive impairment, assessing domain-specific cognitive injury, and predicting plasma biomarker elevation, thus demonstrating EEG’s significant potential for AD early screening. Despite extensive research on AD-related EEG alterations, many studies suffer from small sample sizes and limited EEG indicators. Cassani et al. reported that nearly 50% of EEG-related AD studies from 2010 to 2018 involved fewer than 100 participants (44). This study, in contrast, analyzed 360 samples and 200 EEG indicators in total, incorporating not only traditional absolute and relative power measures but also the recently emphasized power ratio index to minimize variability among individuals across 10 scalp regions. The substantial sample size and diverse EEG indicators improve the reliability of this study and offer significant advantages over comparable studies.

### 4.1 Quantitative EEG indicators effectively screen AD-related cognitive impairment

In the present study, conventional statistical techniques and ML methods were both utilized to evaluate the classification and screening efficacy of various EEG indicators. As shown in Figure 2, the study identified consistent power spectral density patterns among the three groups aligned with prior research, indicating increased slow-wave (delta/theta) power and decreased fast-wave (alpha, beta) power in AD dementia and MCI patients (12, 13, 45). Notably, the left-frontal and right-temporal theta RP emerged as potential EEG markers for distinguishing AD-related cognitive impairment (Table 2). Fernández et al. demonstrated that combining theta dipole density in the left temporal region, as measured by magnetoencephalography, with left hippocampal volume from MRI, could accurately classify 87% of AD and healthy control participants (46). This finding supports the novel observation of increased theta wave RP in the right temporal region in the current study, a previously unreported phenomenon. Additionally, small-sample studies by Fonseca et al. and Jelic et al. showed that indicators such as the absolute and relative power of theta wave in the left frontal region effectively differentiated AD dementia from healthy controls (47, 48). These results align with the present study’s findings on the screening potential of left-frontal theta power, which is probably linked to compensatory upregulation of acetylcholine transferase activity in the frontal lobe and hippocampus of MCI patients, inducing corresponding EEG alterations (49).

Our results showed that conventionally calculated AUCs for individual EEG indicators in classifying AD-related cognitive impairment ranged from 0.70 to 0.78, while the most effective ML classification pattern achieved AUC values of 0.89 for AD dementia, 0.77 for MCI, and 0.87 for healthy controls (Figure 3 and Table 2). These data indicate that ML techniques enhance the differentiation of MCI (mean AUC of 0.64), thus offering significant value for early screening of AD-related cognitive impairment. However, the ML classification rate for MCI in this study did not exceed 0.70. In contrast with previous ML research, studies by Dauwels, Gallego-Jutglà, and Amini reported AUCs above 0.80 for MCI classification (18–20), despite their limited sample sizes (22–65 MCI cases). Small sample sizes can lead to overfitting in ML models, resulting in artificially high classification rates. Based on a larger sample size, Jiao et al.’s study (330 AD dementia cases, 189 MCI cases, and 246 healthy controls) also achieved a lower classification rate for MCI (21). Overall, ML techniques can leverage data more effectively than traditional statistical methods, and future research should increase sample sizes to further enhance qEEG’s screening capacity.

### 4.2 Quantitative EEG indicators have the potential to predict domain-specific cognitive injury and plasma P-tau217

Although extensive research has explored the relationship between EEG indicators and cognitive screening scales like MMSE and MoCA scores (21), studies on the connection between EEG indicators and domain-specific cognitive scores, as well as their predictive capabilities, remain limited. This study investigated the correlation between qEEG indicators and domain-specific cognitive scores and demonstrated significant correlations between the two (Tables 3–5).

In the present study, reductions were observed in alpha2 power, beta1 power, and the alpha1/delta power ratio, while increases were noted in theta power and the theta/beta1 power ratio in participants with domain-specific cognitive impairments, corroborating previous research (22). The AUCs for EEG indicators in predicting domain-specific cognitive impairment ranged from 0.60 to 0.70 (Table 5).

In addition, the study further revealed that specific EEG indicators of affected scalp regions varied across cognitive domains, which emphasized the feasibility for targeted screening of characteristic indicators for each cognitive domain. Moreover, potential overlap in EEG indicators across multiple cognitive domains was noted, such as the theta/beta1 ratio of the left occipital region (Table 5). This overlap likely results from the integrated functioning of cognitive domains through interactions among multiple brain regions to form a large-scale brain network. Thus, even with relatively high AUC, such overlapping EEG indicators might not reliably serve as specific indicators for individual cognitive domains. Consequently, the specific indicators for each domain-specific cognitive function identified in this study, which have not been reported previously, are summarized as follows:

The left-occipital beta1 RP might be a specific correlation index of episodic memory impairment, with an AUC of 0.64 (Table 5). This indicator also shows a significant positive correlation with verbal episodic memory scores (Table 4). Studies have reported a link between beta1 power and episodic memory in patients with cognitive impairment related to psychosis (50) and also in participants with normal cognition (51–53). These results collectively emphasize the essential role of beta waves in episodic memory processes. However, this study identified the most predictive indicator at the occipital electrodes, inconsistent with expectations since the medial temporal lobe and hippocampus are recognized as critical regions for episodic memory encoding and retrieval (54). This discrepancy may be due to the deep location of the hippocampus in the brain, which limits its detection by scalp electrodes. Supporting this, previous scalp EEG studies have reported similar findings (50, 55).

The right-frontal and right-central alpha2 RP might be specific EEG index of non-verbal episodic memory impairment, both with an AUC of 0.70 (Table 5). Also, the RP of alpha2 in the right central region shows a positive correlation with non-verbal episodic memory scores (Table 4). Event-related EEG studies have identified alpha wave desynchronization to be significant in spatial localization and essential for the encoding and retention of spatial information (56). Alpha2 waves have been observed to be involved in the encoding and retrieval of non-verbal episodic memory (56, 57). However, no previous studies have further explored the relationship between scalp electrode and nonverbal situational memory function through ROC curve analysis. Research has suggested that the prefrontal and parietal cortex are involved in the encoding process of episodic memory (58, 59). The electrode of the right central region (C4), located between the surface projection areas of the prefrontal and parietal lobes, might reflect functional changes in these areas.

The left-occipital theta RP (AUC = 0.69) and the right-central theta AP (AUC = 0.65) may serve as specific EEG indicators of executive function impairment (Table 5). Prior event-related EEG research suggests that theta waves convey top-down control signals from the prefrontal cortex to various brain regions and are regarded as EEG indicators of frontal lobe executive control functions (60), with increased theta power linked to longer decision times during conflicts (61). These observations align with the current study’s finding of elevated theta waves in the context of executive function impairment. The study identified the strongest associations in the left occipital and right central regions (C4), which located near the frontal lobe, detecting changes in frontal lobe activity. Previous MRI research has linked declines in executive function to damage in the fronto-occipital fiber bundle, which connects the frontal and occipital regions (62). Consequently, electrodes in the occipital region may reflect executive function by monitoring fronto-occipital fiber bundle activity.

The right-occipital alpha2 AP (AUC = 0.72), left-temporal alpha2 RP (AUC = 0.72), and left-temporal beta1 AP (AUC = 0.73) all had the potential to be specific indicators of visuospatial function impairment (Table 5). Furthermore, left temporal beta1 AP showed a significant positive correlation with visuospatial function scores (Table 4). Prior studies have established a connection between visuospatial attention and alpha waves (56). Moreover, some research indicates that during visuospatial attention tasks, alpha wave power initially decreases, followed by an increase in beta wave power, suggesting a role for beta waves in visuospatial attention as well (63). The occipital lobe, serving as the visual center of the cerebral cortex, is part of the occipital-parietal-medial temporal pathway crucial for visuospatial navigation (64), supporting these observations. Overall, while these domain-specific associations are promising and consistent with some prior literature, they require replication in independent cohorts using more spatially precise neuroimaging methods before they can be interpreted as reflecting specific regional brain dysfunction.

This study also utilized ultra-sensitive single-molecule immunoassay technology to measure plasma P-tau217 concentrations in 105 patients with normal cognition and MCI. The analysis revealed an inverse correlation between plasma P-tau217 levels and right-temporal beta2 RP and right-parietal beta2 RP, respectively. Furthermore, right-parietal beta1 RP was identified as an independent factor associated with elevated plasma P-tau217 concentrations (AUC = 0.68). In 2018, Smailovic et al. conducted a comprehensive study with 637 participants, including healthy controls, MCI, and AD dementia cases, revealing that reduced fast wave (alpha, beta) power correlated with elevated CSF P-tau and T-tau levels (25). Similarly, in 2020, Tanabe et al. identified a negative correlation between alpha peak power and CSF P-tau181 (65). Previous research has demonstrated that plasma P-tau217 exhibits high diagnostic accuracy for both clinical and pathological AD, nearly matching the performance of CSF P-tau217 (27). Building on this, the present study suggests that right-parietal beta RP, which correlates with the plasma biomarker P-tau217, may serve as an early indicator of brain pathological changes in AD, offering substantial potential for early screening of AD-related cognitive impairment.

### 4.3 Limitations

Several limitations should be acknowledged. First, diagnoses were based solely on clinical criteria without biomarker confirmation (CSF or PET), which may introduce etiological heterogeneity, particularly in the MCI group and potentially diminishing the differences between MCI and normal cognitive participants. Thus, this study did not identify EEG indicators with statistically significant differences between MCI and healthy control groups using traditional statistical methods. Second, the effect sizes for domain-specific and P-tau217 predictions were modest (AUCs 0.60–0.70), indicating that current qEEG metrics alone are insufficient for standalone clinical decision-making. Therefore, more cohort studies with larger samples and biomarkers of cerebrospinal fluid or PET-CT are needed to further verify the results of this study. Third, despite FDR correction, the large number of EEG features examined and the multicollinearity of EEG indicators increased the risk of false positives. Fourthly, the spatial pattern based on scalp electrode only reflects the macro-electrophysiological correlation of network activities in different brain regions, not the direct mapping of local neural sources, and its potential neural mechanism still needs to be verified by neuroimaging techniques such as high-density EEG source localization or functional magnetic resonance imaging. Finally, as a single-center cross-sectional study, longitudinal and multicenter validation is needed to confirm the EEG indicators of AD-related cognitive impairment identified in the present study.

## 5 Conclusion

In summary, this study demonstrates that quantitative EEG indicators are not only a promising screening tool for AD-related cognitive impairment but also potential predictors of domain-specific cognitive impairments (including episodic memory, executive function, and visuospatial abilities) and plasma P-tau217 levels. However, current performance metrics (AUCs 0.60–0.87) indicate that qEEG is not yet sufficient for standalone diagnostic use. These findings suggest that qEEG holds promise as a non-invasive, complementary screening approach for AD-related cognitive impairment, and further external validation in prospective, multicenter cohorts is required before implementation in community or primary care settings.

## Data Availability

The raw data supporting the conclusions of this article will be made available by the authors, without undue reservation.
